# Say ‘no’ to carcinogen as contraception alternative 

**DOI:** 10.4102/phcfm.v4i1.424

**Published:** 2012-06-29

**Authors:** Karen Malec

**Affiliations:** 1 Coalition on Abortion/Breast Cancer, USA

## Abstract

A response to Correspondence to ‘Dienye PO, Gbeneol PK. Contraception as a risk factor for urinary tract infection in Port Harcourt, Nigeria: A case control study. Afr J Prm Health Care Fam Med. 2011;3(1), Art. #207, 4 pages. doi:10.4102/phcfm.v3i1.207

## To the editor:

In their study, *‘Contraception as a risk factor for urinary tract infection in Port Harcourt, Nigeria: A case control study’,*^1^ the authors found a statistically significant association of urinary tract infections with use of barrier methods of contraception. In their conclusions they wrote: “Women who use the barrier methods could be advised to consider alternative methods, such as oral contraceptives.”

In 2005 the World Health Organization added combined oral contraceptives (COCs) and combined hormone replacement therapy to its list of group 1 carcinogens, citing the former as a risk factor for cancers of the breast, liver and cervix.^2^ COCs appear on the same list as tobacco, asbestos, cadmium and benzene.

In 2006 Mayo Clinic Proceedings published a meta-analysis whose authors reported a statistically significant 44% increased risk of premenopausal breast cancer amongst users of oral contraceptives before first full-term pregnancy.^3^

In two studies oral contraceptive use has been strongly linked with the deadly triple-negative breast cancer, which occurs most often amongst young women under age 50 and African American women.^4,5^

Drs Dienye and Gbeneol reported, ‘There was about a three-fold increased risk of the development of urinary tract infection amongst patients who were on contraceptives compared to non-users.’ Since their study showed non-use to be superior to any form of contraception in terms of Urinary Tract Infection (UTI) risk and since Natural Family Planning is as effective as chemical contraception, with none of the cancer risk, why not recommend Natural Family Planning to patients? 

A carcinogen should not be recommended as an alternative to barrier methods of contraception. 

## Dienye PO, Gbeneol PK respond:

Combined hormonal contraceptives consist of an oestrogen and a progestogen, and act primarily by preventing ovulation through inhibition of follicle-stimulating hormone and luteinising hormone. The progestogen component also renders the cervical mucous relatively impenetrable to sperm and reduces the receptivity of the endometrium to implantation. These mechanisms render combined hormonal contraceptives very effective in prevention of pregnancy. Annual failure rates vary between 0.02% (2 per 10 000 women/year) when full adherence to instructions for use is assumed.^6,7^ 

According to Blackburn et al.^8^ more than 100 million women – an estimated 10% of all women of reproductive age – currently use combined hormonal contraceptives, a large majority of which are in the form of oral preparations. They were the most widely used method of contraception among married women in two-thirds (44/68) of developing countries. Current use of these drugs is greatest in developed countries (16%) and is lower in developing countries (6%). In developing countries 32% of women were estimated to have ever used hormonal contraception. Overall the use of combined hormonal contraception is increasing, but there is extreme variability between countries. In many countries these preparations are mainly used by women of younger age and higher level of education, and who have greater access to health care. The popularity of COCs has been attested to by Bongaarts et al.,^9^ who projected that in the developing world their use would double between 1993 (11% of women) and 2015 (22%). This trend is attributed to improved access, changes in the characteristics of users with better education, a desire for smaller families, and new and improved technology. 

Inclusion by the WHO in 2005 of COCs and combined hormone replacement therapy on its list of group 1 carcinogens, citing the former as a risk factor for cancers of the breast, liver and cervix,^10^ is a recognised fact. Other major non-cancerous risks of COC use include ischaemic stroke, venous thrombo-embolism and myocardial infarction, but these are rare events in women of childbearing age, and the attributable risks are very small.^11,12^ Although the carcinogenic effect of oral contraceptives has been reported,^13^ the relative risk is small and the absolute risk (excess breast cancers due to COC exposure) is very small. For example, the Oxford pooled analysis estimates that the excess number of cases of breast cancer expected to be diagnosed up to 10 years after discontinuation of COC use among 10 000 European or North American women is 0.5 cases for COC use from age 16 to 19 years, 1.5 cases for COC use from age 20 to 24 years, and 4.7 cases for COC use from 25 to 29 years. These cases are also likely to be clinically localised.

There are overwhelming benefits to using these drugs. Firstly, COCs are extremely effective in preventing pregnancy when used correctly.^11^ Additionally, the International Agency for Research on Cancer has reported that COCs decrease the risk of ovarian and endometrial cancer, and there is accumulating evidence that they may lower the risk of colorectal cancer.^13^ Finally, there is a growing number of non-contraceptive health benefits associated with COCs, including relief from menstrual disorders, reduced risk of pelvic inflammatory disease, benign breast disease, uterine leiomyomas and ovarian cysts, and improved bone mineral density.^11^ In the locality of the study it is easy to screen for breast and cervical cancer, considering the fact that such procedures are not invasive. Screening for carcinoma of the endometrium and colon is not commonly done due to non-availability of equipment and skilled manpower. The protective property of COCs against colonic and endometrial cancer justifies its prescription in the locality.

It may therefore be advisable to closely follow the epidemiology of COC use and health outcomes, given the widespread use of these agents and their high potential to impact women’s health in both a beneficial and a deleterious manner. 

We conclude that although we made the recommendation of COC use, the discretion of the prescribing physician is very important. It also has to be noted that as long as the WHO has not pronounced these drugs as banned, criticising their prescription or recommendation of their prescription appears parochial and should not be encouraged. 
